# Comparison of the Immunogenicity and Protective Efficacy of Various SARS-CoV-2 Vaccines among Healthcare Workers: Are Our White Coat Armies Protected?

**DOI:** 10.3390/vaccines10050642

**Published:** 2022-04-20

**Authors:** Lina Souan, Maher A. Sughayer, Maha M. Abualhour, Mahmoud Siag, Sara Al-Badr, Tareq Al-Atrash

**Affiliations:** Department of Pathology & Laboratory Medicine, King Hussein Cancer Center, Amman 11941, Jordan; lsouan@khcc.jo (L.S.); ma.15153@khcc.jo (M.M.A.); msiag@khcc.jo (M.S.); sa.14588@khcc.jo (S.A.-B.); tatrash@khcc.jo (T.A.-A.)

**Keywords:** COVID-19, SARS-CoV-2, antibody titer, neutralizing antibody, IgG, healthcare workers

## Abstract

Background: The effective immunization of healthcare workers (HCWs) plays a vital role in preventing the spread of SARS-CoV-2 infection during the coronavirus disease 2019 (COVID-19) pandemic. There is limited data on the immune response to vaccination among HCWs. We aim to determine seroprevalence rates and neutralizing IgG antibody response to various immunizations among HCWs. Methods: This study was conducted between July and September 2021, in which blood samples were obtained from HCWs and SARS-CoV-2 IgG neutralizing antibodies were measured. Data regarding vaccination status with Pfizer/BioNTech, Sinopharm, or AstraZeneca vaccines, occupation, and prior COVID-19 infection were analyzed. Results: COVID-19 infection post-vaccination was associated with higher mean antibody titers, regardless of vaccine type. Pfizer/BioNTech vaccination produced higher mean antibody titers for HCWs with prior COVID-19 infection (*p* < 0.00001) than other types of vaccines. Although 96% of HCWs were vaccinated, 3% were seronegative. For HCWs who were seropositive, there were no significant differences between the mean antibody titers when comparing occupations and blood indices. Conclusion: Awareness of the immunity status of HCWs is key to protecting this important group against SARS-CoV-2, especially those without prior COVID-19 infection. Further public health efforts regarding booster vaccination for HCWs are crucial to provide necessary antibody protection.

## 1. Introduction

Vaccines against SARS-CoV-2 have been developed in an unexpectedly brief period [1,2]. Data, updated to 24 October 2021, reported that 23 vaccines were each approved by at least one country and have been granted emergency use authorization or made available for use outside of clinical trials via any pathway by a regulatory agency, a national authority, or another entity [3]. Studies showed that vaccines contributed to the control of the COVID-19 pandemic worldwide, and may have provided a degree of herd immunity [4,5,6].

In Jordan, the first case of COVID-19 appeared in March 2020, and from that date, the government adopted restricted measurements and rules related to travel, education, and religious and social events, in addition to working within numerous industries [7,8,9]. This resulted in a mild first wave with limited deaths and according to the “Situation Report 83”, which was issued by the WHO on 12 April 2020, Jordan was only classified as a “Cluster of cases” [10]. Unfortunately, the country was struck by a second aggressive wave in September 2020, resulting in many deaths and losses. Many physicians and healthcare workers (HCWs) lost their lives because of the infection while performing their duties [11]. Referring to a survey conducted between 22 July and 15 August 2020, 54 had the infection of which 23 died [12]. A recent report by the WHO showed that between January 2020 and May 2021 there were 6643 HCW deaths worldwide out of the 3.45 million COVID-19-related deaths [13].

In most communities, HCWs are exposed to the virus at a greater level than any other society members and may be considered at an elevated risk of infection. Their role in the chain of transmission is essential, through which they help in the control and prevention of the spread of COVID-19 infection. For these reasons, vaccination strategies of many countries including Jordan focused on treating HCWs as a priority group [14]. It was reported that Jordan was among the first 40 countries to be vaccinated, the campaign was launched on 13 January 2021 beginning with healthcare workers, people with chronic diseases, and those over the age of 60 [15].

Jordan Food and Drug Administration (JFDA) approved the use of the following vaccines; Pfizer/BioNTech (Multinational), Sinopharm (Beijing, China), AstraZeneca (Oxford, UK), Johnson & Johnson (Beerse, Belgium), and Sputnik V (Gamaleya, Russia) [16]. According to the latest statistics from the Jordanian Ministry of Health, there are 4,556,988 who received one dose, while 4,168,651 received two doses [17]. Sughayer et al. reported that the seroprevalence of neutralizing antibodies among healthy blood bank donors at King Hussein Cancer Center (KHCC) was 27.4% in early February 2021 [18].

In this study, we enrolled 510 HCWs from KHCC who either received or did not receive COVID-19 vaccination. We aimed to determine the correlation between clinicopathological characteristics and production of anti-SARS-CoV-2 neutralizing IgG antibody and its titer levels in those who received the vaccine or those who had been exposed to the SARS-CoV-2 virus and had subclinical infections (asymptomatic) without being vaccinated. In addition, we investigated if there is any correlation between seroprevalence rates and neutralizing IgG antibodies titers within the HCWs demographics.

## 2. Materials and Methods

A prospective cross-sectional seroepidemiological study was conducted over three months (from July to September 2021) at KHCC. After the approval of the Institutional Review Board (IRB) at KHCC, 510 health care workers (HCWs) were randomly recruited, filled out a consent form, and the information related to the study was collected in a data collection sheet. Serum samples were collected (3–5 mL) then centrifuged at 4300 rcf for 5 min then stored at −80 degrees Celsius until analyzed. Frozen samples were thawed gently by transferring the samples to a 4 degrees Celsius refrigerator for 24 h before analysis. The samples were then vortexed before sample processing and analysis.

Samples were tested using the SARS-CoV-2 IgG II Quant assay. The test is a chemiluminescent microparticle immunoassay for the quantitative determination of IgG antibodies to SARS-CoV-2, including neutralizing antibodies, to the receptor-binding domain (RBD) of the S1 subunit of the spike protein of SARS-CoV-2 in serum and plasma from individuals who are suspected to have had coronavirus disease (COVID-19) or in serum and plasma of individuals that may have been infected by or vaccinated against SARS-CoV-2 (Abbott Architect SARS-CoV-2 IgG with ARCHITECT i1000SR analyzer; Abbott Laboratories, Chicago, IL, USA) according to the manufacturer’s instructions [19]. The Abbott SARS-CoV-2 IgG II Quant assay has a measuring range of 21–40,000 AU/mL, with ≥50 AU/mL considered positive. The assay has 99.35% sensitivity and 99.6% specificity with 100% (86/86) positive agreement with the plaque reduction neutralization test (PRNT); 95% CI = 95.72. Plaque reduction neutralization tests (PRNT) are used to quantify the titer of neutralizing antibodies for a virus [19].

An additional dipotassium EDTA–anticoagulated peripheral blood sample was collected from all participants and analyzed on the Beckman Coulter-UniCel DxH 800 Coulter cellular analyzer (California, CA, USA) to measure clinicopathological characteristics such as hemoglobin level, white blood cell, lymphocyte, platelets, and neutrophils counts. Laboratory tests’ normal ranges are listed in Appendix A. If the test result was normal, the HCW was placed under the “Normal” group, otherwise, the HCW was placed under the “Not Normal” group.

The COVID-19 vaccine was administered to HCWs, which was provided by the Jordanian Ministry of Health (MOH) with no control in opting for the type of vaccine administered to them. All HCWs took the first and the second dose from the same type of vaccine.

Participants’ characteristics and vaccine information were presented as counts and percentages, such as vaccine type, in addition, to mean and range to describe age and other continuous factors. Comparison between vaccination rates, types, and outcomes, according to all factors, were carried out using t-test, one-way ANOVA from summary data, or Chi-square test as appropriate using the Analysis of Variance from Summary Data online website https://statpages.info/anova1sm.html (accessed on 20 January 2022). In addition to analysis performed using SAS version 9.4 (SAS Institute Inc., Cary, NC, USA).

## 3. Results

### 3.1. Neutralizing IgG Seroprevalence and Titer Rates Correlation with HCWs Demographics

In the studied group, there were 255 males (255/474 = 53%) who had the COVID-19 vaccination and their mean titer level was 6687.4 (11.4–40,000) AU/mL, compared to 219 (219/474 = 46.2%) females with a mean titer level of 5827.2 (0.0–40,000) AU/mL; there was a significant difference between both genders in the numbers of vaccinated vs. non-vaccinated participants (*p*-value < 0.05) but there was no significant difference in the mean titer levels between both genders (Table 1 and Table 2). The mean age of the COVID-19 vaccinated participants was 35 (20.3–70.4) years while in the non-vaccinated group the mean age was 33.1 (24.9–61.4) years old. There was no significant difference between both groups (*p*-value 0.315).

To investigate the relationship between the seroprevalence rates and the vaccination status and history of previous COVID-19, we analyzed data reported by the HCWs at the time of sample collection on whether they recalled having a COVID-19 infection before participating in the study. Data showed that more than half of the HCWs who tested positive for the neutralizing IgG antibodies in the unvaccinated group have had COVID-19 infection before participating in the study: 8 (61.53%), while 5 (38.46%) did not report that they had previous COVID-19 infection. On the other hand, 209 (46.23%) of seropositive-vaccinated HCW have had COVID-19 infection before participating in the study while 242 (53.53%) did not report that they had previous COVID-19 infection and this difference between COVID-19 infection and no infection had no significant effect on the seroconversion to positive status among vaccinated and unvaccinated HCWs (Table 3).

Out of the 510 HCWs, 25 participants had insufficient data. Of the remaining 485 HCWs, there were 466 (95.88%) vaccinated and 19 (3.9%) unvaccinated at the time of study closure. Among the vaccinated group, there were 452 (96.99%) who tested positive for neutralizing IgG antibodies and 14 (3%) were negative. While in the unvaccinated group, there were 13 (68.42%) who tested positive for neutralizing IgG antibodies and 6 (31.57%) were seronegative. These data showed a significant difference between vaccinated and unvaccinated groups in the seroprevalence rates of anti-SARS-CoV-2 neutralizing IgG antibodies (*p*-value < 0.0001) (Table 3).

### 3.2. The Effect of COVID-19 Infection on Neutralizing IgG Antibodies Titer in HCWs

We analyzed the possible effect of COVID-19 infection before or after vaccination on neutralizing IgG antibodies titer. Our data demonstrated that there was a significant difference in neutralizing IgG antibodies titer between HCWs who had COVID-19 infection and the ones who did not have an infection (Table 4) and this difference was seen in all vaccine types (Table 5). 

**Figure 1:**
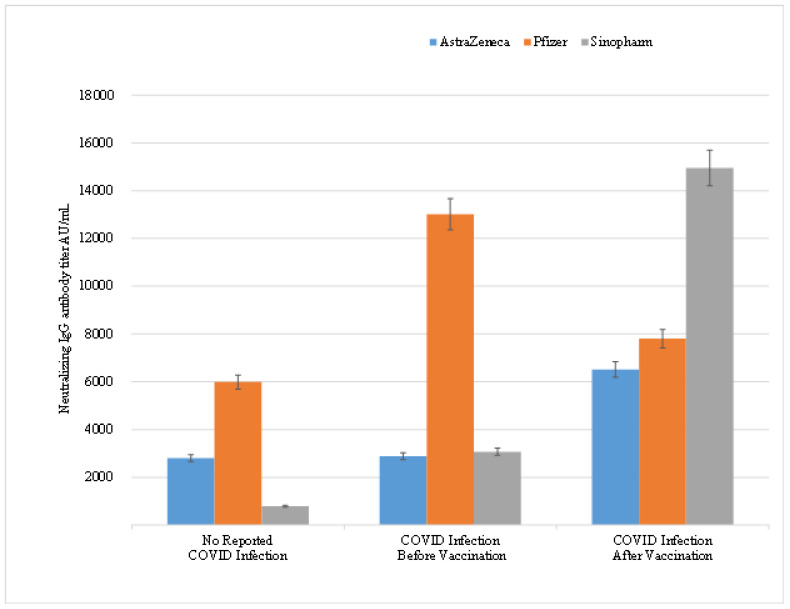
Correlation between the times of acquiring COVID-19 infection on the mean titer levels of neutralizing IgG antibodies in HCWs.

Further analysis of the time of acquiring the COVID-19 infection and its effect on neutralizing antibody titer in vaccinated HCWs with different types of vaccines, revealed that COVID-19 infection had a significant positive effect resulting in an increase in neutralizing IgG titer in HCWs infected with COVID-19 before vaccination with Pfizer/BioNTech and Sinopharm vaccines. The mean antibody titer was 5981.83 AU/mL in individuals who did not report COVID-19 infection before vaccination compared to 13,018.12 AU/mL in HCWs who reported infection prior to vaccination (*p*-value < 0.00001) (Table 6). Likewise, HCWs’ vaccinated with Sinopharm showed a significant increase from 788 AU/mL to 3074.9 AU/mL (*p*-value < 0.05) between the same two aforementioned groups. Interestingly, HCWs who reported COVID-19 infection after vaccination showed an increase in the mean titer levels of neutralizing antibodies regardless of the type of vaccine demonstrating the booster or adjuvant effect of the COVID-19 infection on the vaccine through increasing the mean titer of the neutralizing antibodies. This reported increase was more significant in Sinopharm vaccinated HCWs (*p*-value < 0.00001) compared to Pfizer/BioNTech vaccinated (*p*-value = 0.97) or AstraZeneca vaccinated HCWs (*p*-value was 0.44) in (Figure 1).

### 3.3. Neutralizing IgG Antibody Titer Correlation with the Occupation of HCW

HCWs’ occupation was grouped according to the extent of exposure to patients. The HCWs were grouped into 6 groups as follows: laboratory (included lab directors, supervisors, research assistants, and medical technologists), nursing, medical staff (physicians, dentists, medical students, clinical research coordinators, radiology, and endoscopy technicians), medical support staff (dental technicians, endoscopy technologists, radiology technologists, and research assistants), pharmacists, support staff (hospitality services, housekeeping, porters, transportation) while the remaining occupations (such as administration, engineers, maintenance technicians, finance, human resources, information technologists, psychosocial, quality safety and environmental health officers, material and management personnel, and security), were gathered under one group called others. The seroconversion rates and mean titer values among vaccinated and non-vaccinated HCWs grouped according to their occupation are illustrated in Table 7.

**Figure 2:**
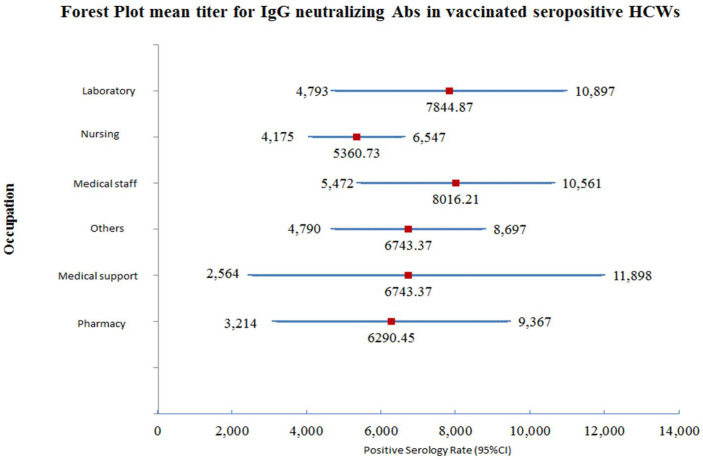
Forest plot for the mean titer and 95% CI for neutralizing antibody IgG antibody titer among vaccinated-seropositive HCWs.

Out of the 510 participants, 24 participants had insufficient data and out of the remaining 486 participants, there were 43 who were laboratory pathologists and medical technologists. Their mean titer was 7844.87 AU/mL, with a 95% confidence interval (4793.11–10,896.62). The mean titer of the neutralizing IgG antibody in 180 nursing staff was 5360.73 AU/mL, with a 95% confidence interval (4174.80–6546.66). The number of medical staff who were tested was 98 and their mean titer of neutralizing IgG antibody was 8016.22 AU/mL, with a 95% confidence interval (5471.64–10,560.80). There were 22 medical support staff participants and their mean titer antibody after vaccination was 7231.35 AU/mL, with a 95% confidence interval (2564.22–11,898.47). The number of participating pharmacists was 30, and the mean titer of the protective neutralizing IgG antibody developed was 6290.45 AU/mL, with a 95% confidence interval (3214.32–9366.58). The remaining HCWs in the study were 113, and their mean titer of protective neutralizing IgG antibody was 6743.37 AU/mL, with a 95% confidence interval (4790.05–8696.70). A forest plot for the mean titer and range for neutralizing antibody titer is illustrated in Figure 2.

### 3.4. Correlation between Clinicopathological Characteristics and Production of Neutralizing Antibody Titer

Among 472 participants, there was no significant correlation between the mean titer of neutralizing IgG antibody generated after vaccination with different types of vaccines and the blood group, or hemoglobin level (HB), white blood cell count (WBCs), lymphocyte count, platelet count, or neutrophil count (Table 8).

## 4. Discussion

In this study, we describe the seroprevalence rates and anti-SARS-CoV-2 neutralizing IgG antibody titer levels for HCWs based on demographics, clinicopathological characteristics, and pre-and post-COVID-19 infections at a cancer center from July to September 2021. Our data showed that the three types of vaccines (Pfizer/BioNTech, Sinopharm, and AstraZeneca) included in this study induced an appropriate neutralizing IgG immune response. In our study, more female-HCWs took the SARS-CoV-2 vaccine compared to males; however, there was no significant difference in the mean titer levels between males and females as was shown in other studies [20]. Badano MN et al. found higher titer levels in Sinopharm vaccinated female HCWs, but further studies are recommended to evaluate any similar trends in our population [21].

Full compliance to vaccination was seen among laboratory, medical support staff, and pharmacy (100%) followed by nursing, then the “others” group while the least compliance rates were reported among the physicians’ group 96.1%, 94.53%, and 94.29%, respectively. Concerning the seroconversion rates, our data showed that the highest seroconversion rate was among pharmacists followed by nursing staff, physicians, laboratory, others, and finally the medical support staff group. On the other hand, the highest anti-SARS-Co-V2 neutralizing antibodies titer was seen among the vaccinated positive-seroconverted physicians followed by laboratorians, others, medical support staff, pharmacy, and lastly, was the nursing group. This pattern of seroconversion rates might serve as a roadmap for future booster shot vaccine campaigns.

Although our data showed no relation between antibody titers and blood indices, it demonstrated that COVID-19 vaccination for HCWs is effective in producing neutralizing IgG antibodies against SARS-CoV-2 infection, notably with a significantly higher titer in HCWs who reported SARS-CoV-2 infection post-vaccination across all vaccine types studied. Furthermore, specifically, for HCWs receiving Pfizer and Sinopharm vaccines, there was a higher titer for those who had self-reported COVID-19 infections prior to vaccination compared to those who were infected with COVID-19. This was true for the three studied vaccines and confirmed previously published data [22]. Our findings are suggestive that COVID-19 vaccination increases existing immunity in HCWs and provides additional protection against further infection. However, among vaccinated HCWs, 3% were negative for neutralizing IgG antibodies with no reported previous COVID-19 infection, raising concern for inadequate protection against SARS-CoV-2 infection although the average age range for this group was 43 years and they were all healthy with no reported illnesses or medications were taken at the time of sample testing. Given these findings, we conclude that booster vaccination campaigns are necessary to provide HCWs with the immunity needed to fight this deadly virus. Our results suggest that priority for booster vaccines should be given to HCWs who did not have prior COVID-19 infections, as all vaccinated HCWs in our study who did not seroconvert had no reported history of COVID-19 infection.

The limitation of this study is that COVID-19 infection was reported based on HCW self-reporting and not on precise PCR testing. It is difficult to say that the HCWs who reported negative COVID-19 infection, might have been asymptomatically infected with the virus without knowing it. Therefore, to better understand the effect of COVID-19 infection on vaccinated individuals, controlled prospective studies are needed for HCWs who are PCR tested for COVID-19 infection before and after vaccination. The increase in the antibody titer might also be due to variation in the time of acquiring the infection and taking the blood sample. The involvement of any undetected confounders of the vaccine-induced humoral response cannot be completely excluded.

Our data raise concerns about whether our primary defense line workers in health care have adequate protective immunity following vaccination for SARS-CoV-2. Taken together, these findings suggest that evaluation of vaccine-induced immunity in HCWs should be examined and booster shots are recommended especially among individuals who have no history of COVID-19 infection.

The results may lead to a better understanding of COVID-19’s spread across Jordanian healthcare facilities, identify asymptomatic infections, evaluate and measure vaccine-induced immunity among HCWs, and whether our HCWs, as our first line of defense, are adequately protected against COVID-19 infection.

## Figures and Tables

**Table 1:** Total number and percentage of vaccinated and non-vaccinated HCWs.

Label	Total N = 510	COVID Vaccinated N = 474 (92.9%)	Not Vaccinated N = 36 (7.1%)	*p*-Value
F	229 (44.9%)	219 (95.6%)	10 (4.4%)	0.0321
M	281 (55.1%)	255 (90.7%)	26 (9.2%)	

**Table 2:** Mean titer (range) AU/mL for anti-SARS-CoV2 neutralizing IgG antibodies in vaccinated HCWs for males and females.

Value	Mean Titer (Range) AU/mL	*p*-Value
F	5827.2 (0.0, 40,000)	0.762
M	6687.4 (11.4, 40,000)	

**Table 3:** COVID-19 infection rate before sample testing among vaccinated and non-vaccinated HCWs and their seroconversion status.

Vaccinated HCWs N = 466 95.88%	**Seropositive/Immune**		* *p*-Value	**Negative**	
	452 (96.99%)			14 (3.00%)	
	* Previous COVID-19 infection	* No reported Infection		Previous COVID-19 Infection	No Reported Infection
	209 (46.23%)	242 (53.53%)	0.12	0 (0%)	14 (100%)
Unvaccinated HCWs N = 19 3.90%	**Seropositive/Immune **		** *p*-value	**Negative**	
	13 (68.42%)			6 (31.57%)	
	** Previous COVID-19 infection	** No reported Infection		Previous COVID-19 infection	No reported Infection
	8 (61.53%)	5 (38.46%)	0.41	0 (0%)	6 (100%)

* Significance was calculated between the vaccinated-seropositive HCWs with reported previous COVID-19 infection and the vaccinated-seropositive HCWs with NO reported previous COVID-19 infection. ** Significance was calculated between the Unvaccinated-seropositive HCWs with reported previous COVID-19 infection and the Unvaccinated-seropositive HCWs with NO reported previous COVID-19 infection..

**Table 4:** The effect of COVID-19 infection on the mean titer of neutralizing IgG antibodies in vaccinated HCWs regardless of the type of vaccine and the time of infection.

COVID-19 Infection.	Mean Titer (Range) AU/mL	*p*-Value
No	3774.6 (0.0–40,000)	<0.0001
Yes	9414.1 (131.6–40,000)	

**Table 5:** The effect of COVID-19 infection on the mean titer of neutralizing IgG antibodies in HCWs vaccinated with three main types of vaccines.

Vaccine Type	COVID-19 Infection (Yes/No)	Mean (Min, Max) Titer AU/mL	*p*-Value
AstraZeneca	No	2805 (38.1–27,965)	0.005
	Yes	3019 (251.0–9006)	
Pfizer/BioNTech	No	4702 (0.0–36,213)	<0.0001
	Yes	10,925 (760.9–39,441)	
Sinopharm	No	778.2 (11.4–17,813)	<0.0001
	Yes	3261 (131.6–31,159)	

**Table 6:** The effect of the time of acquiring COVID-19 infection on the mean titer of neutralizing IgG antibodies in HCWs vaccinated with three main types of vaccines.

Vaccine Type		No Reported COVID-19 Infection	COVID-19 Infection before Vaccination	COVID-19 Infection after Vaccination	*p*-Value
AstraZeneca	Total number of HCWs	59	33	2	
Total N = 94	Titer Mean AU/mL	2805.117	2891.245	6512.25	0.443
	(95% CI) LL-UL	1549.3–4060.9	2185.61–3596.87	1333.836–11,690.66	
Pfizer/BioNTech	Total number of HCWs	117	128	2	
Total N = 248	Titer Mean AU/mL	5981.834	13,018.115	7802.15	0.00
	(95% CI) LL-UL	4359.53–7604.13	10,962.22–15,074.01	−23,642.5–39,246.83	
Sinopharm	Total number of HCWs	73	38	4	
Total N = 116	Titer Mean AU/mL	787.9931507	3074.884211	14,947.45	0.00
	(95% CI) LL-UL	148.55–1427.43	632.87–5516.899	−12,034.3–41,929.2	

**Table 7:** The seroconversion rates among vaccinated and unvaccinated HCWs according to their occupation with a history of COVID-19 infection at any time prior to the time of sampling.

Occupation	Vaccinated, Seropositive HCWs			Unvaccinated, Seropositive HCWs	
	Previous COVID-19 Infection *	No Reported Infection *	*p*-Value *	Previous COVID-19 Infection	No Reported Infection
Laboratory	17 (41.46%)	23 (56.09%)	0.34	0 (0.00%)	0 (0.00%)
Nursing	87 (50.28%)	86 (49.71%)	0.94	3 (75%)	1 (25%)
Medical Staff	45 (50%)	45 (50%)	1	2 (50%)	2 (50%)
Others	40 (40%)	60 (60%)	0.05	3 (60%)	2 (40%)
Support Staff	10 (50%)	10 (50%)	1	0 (0.00%)	0 (0.00%)
Pharmacy	10 (35.71%)	18 (64.28%)	0.13	0 (0.00%)	0 (0.00%)

* Significance was calculated between the vaccinated-seropositive HCWs with reported previous COVID-19 infection and the vaccinated-seropositive HCWs with NO reported previous COVID-19 infection.

**Table 8:** Correlation between clinicopathological characteristics and mean titer and range of neutralizing IgG antibodies in vaccinated HCWs.

Variant		Mean Titer (Range) AU/mL	*p*-Value
Blood Group	A	6217.8 (15.9–40,000)	0.241
	AB	8276.9 (354.7–40,000)	
	B	6082.8 (33.6–40,000)	
	O	6157.5 (0.0–40,000)	
HB	Normal	6051.0 (0.0–40,000)	0.368
	Not Normal	7386.9 (39.4–40,000)	
WBCs	Normal	6221.0 (11.4–40,000)	0.492
	Not Normal	3607.2 (23.3–18,178)	
Lymphocytes 10^3^/uL	Normal	6350.5 (11.4–40,000)	0.106
	Not Normal	4996.3 (23.3–40,000)	
Platelets	Normal	6120.5 (11.4–40,000)	0.491
	Not Normal	5837.9 (74.4–40,000)	
Neutrophils 10^3^/uL	Normal	6254.6 (11.4–40,000)	0.571
	Not Normal	5920.1 (15.9–23,779)	

## Data Availability

The data presented in this study are available by request from the corresponding author. The data are not publicly available for ethical reasons.

## Appendix A

**Table A1:** Laboratory tests normal ranges.

Test	Age		Gender	Unit	Lower Limit of Normal Range	Upper Limit of Normal Range
	Lower	Upper				
Hemoglobin (HB)	18	99	Female	g/dL	12	16
	18	99	Male	g/dL	13	18
White blood cell count (WBCs)	18	99	Both	10^3^/uL	4	11
Lymphocytes	18	99	Both	10^3^/uL	0.9	3.4
Neutrophils	18	99	Both	10^3^/uL	2.2	7.1
Platelets	18	99	Both	10^3^/uL	150	400

## References

[1] Polack F.P., Thomas S.J., Kitchin N., Absalon J., Gurtman A., Lockhart S., Perez J.L., Pérez Marc G., Moreira E.D., Zerbini C. (2020). Safety and Efficacy of the BNT162b2 mRNA Covid-19 Vaccine. N. Engl. J. Med..

[2] Voysey M., Clemens S.A.C., Madhi S.A., Weckx L.Y., Folegatti P.M., Aley P.K., Angus B., Baillie V.L., Barnabas S.L., Bhorat Q.E. (2021). Safety and efficacy of the ChAdOx1 nCoV-19 vaccine (AZD1222) against SARS-CoV-2: An interim analysis of four randomised controlled trials in Brazil, South Africa, and the UK. Lancet.

[3] Team MCVT (2021). COVID-19 Vaccine Tracker. JORDAN: 4 Vaccines Approved for Use in Jordan. McGill University McGill University Interdisciplinary Initiative in Infection and Immunity (MI4). https://covid19.trackvaccines.org/country/jordan/.

[4] Au J. (2021). Higher Vaccination Rate Predicts Reduction in SARS-CoV-2 Transmission across the United States. medRxiv.

[5] Ferrari D., Mangia A., Spano M.S., Zaffarano L., Vigano M., Di Resta C., Locatelli M., Ciceri F., De Vecchi E. (2021). Quantitative serological evaluation as a valuable tool in the COVID-19 vaccination campaign. Clin. Chem. Lab. Med..

[6] Glampson B., Brittain J., Kaura A., Mulla A., Mercuri L., Brett S.J., Aylin P., Sandall T., Goodman I., Redhead J. (2021). Assessing COVID-19 Vaccine Uptake and Effectiveness Through the North West London Vaccination Program: Retrospective Cohort Study. JMIR Public Health Surveill..

[7] Alqutob R., Al Nsour M., Tarawneh M.R., Ajlouni M., Khader Y., Aqel I., Kharabsheh S., Obeidat N. (2020). COVID-19 Crisis in Jordan: Response, Scenarios, Strategies, and Recommendations. JMIR Public Health Surveill..

[8] AlQutob R., Moonesar I.A., Tarawneh M.R., Al Nsour M., Khader Y. (2020). Public Health Strategies for the Gradual Lifting of the Public Sector Lockdown in Jordan and the United Arab Emirates During the COVID-19 Crisis. JMIR Public Health Surveill..

[9] Al-Tammemi A.B. (2020). The Battle Against COVID-19 in Jordan: An Early Overview of the Jordanian Experience. Front. Public Health.

[10] World Health Organization (2020). Coronavirus Disease 2019 (COVID-19) Situation Report—83.

[11] Luck T. New Lockdown Measures for Jordan as Nine Doctors Die in Covid-19 Surge, *The National*, 1 November 2020. https://www.thenationalnews.com/world/mena/new-lockdown-measures-for-jordan-as-nine-doctors-die-in-covid-19-surge-1.1103558.

[12] Erdem H., Lucey D.R. (2021). Healthcare worker infections and deaths due to COVID-19: A survey from 37 nations and a call for WHO to post national data on their website. Int. J. Infect. Dis..

[13] WHO The Impact of COVID-19 on Health and Care Workers: A Closer Look at Deaths. World Health Organization: Geneva, Switzerland, September 2021. https://apps.who.int/iris/bitstream/handle/10665/345300/WHO-HWF-WorkingPaper-2021.1-eng.pdf.

[14] Mortgat L., Verdonck K., Hutse V., Thomas I., Barbezange C., Heyndrickx L., Fischer N., Vuylsteke B., Kabouche I., Ariën K.K. (2021). Prevalence and incidence of anti-SARS-CoV-2 antibodies among healthcare workers in Belgian hospitals before vaccination: A prospective cohort study. BMJ Open.

[15] AFP Jordan Launches Covid Vaccination Campaign. *FRANCE 24*, 13 January 2021. https://medicalxpress.com/news/2021-01-jordan-covid-vaccination-campaign.html.

[16] Jordanian Ministry of Health (2021). Vaccines and Medicines for Corona Virus.

[17] COVID-19 Statistical Report—Amman, Jordan 2021. https://corona.moh.gov.jo/en.

[18] Sughayer M.A., Mansour A., Al Nuirat A., Souan L., Ghanem M., Siag M. (2021). Dramatic rise in seroprevalence rates of SARS-CoV-2 antibodies among healthy blood donors: The evolution of a pandemic. Int. J. Infect. Dis..

[19] ABBOTT AdviseDx SARS-CoV-2 IgG II. Abbott Ireland: ABBOTT; March 2021. https://www.fda.gov/media/146372/download.

[20] Brehm T.T., Thompson M., Ullrich F., Schwinge D., Addo M.M., Spier A., Knobloch J.K., Aepfelbacher M., Lohse A.W., Lütgehetmann M. (2021). Low SARS-CoV-2 infection rates and high vaccine-induced immunity among German healthcare workers at the end of the third wave of the COVID-19 pandemic. Int. J. Hyg. Environ. Health.

[21] Badano M.N., Sabbione F., Keitelman I., Pereson M., Aloisi N., Colado A., Ramos M.V., Wilczyñski J.M.O., Pozner R.G., Castillo L. (2022). Humoral response to the BBIBP-CorV vaccine over time in healthcare workers with or without exposure to SARS-CoV-2. Mol. Immunol..

[22] Dashdorj N.J., Wirz O.F., Roltgen K., Haraguchi E., Buzzanco A.S., Sibai M., Wang H., Miller J.A., Solis D., Sahoo M.K. (2021). Direct comparison of antibody responses to four SARS-CoV-2 vaccines in Mongolia. Cell Host Microbe.

